# Elucidation and functional characterization of the biosynthetic pathway of the natural sweetener phyllodulcin in *Hydrangea macrophylla*

**DOI:** 10.1038/s41598-026-47892-x

**Published:** 2026-04-09

**Authors:** Goutham Padmakumar Sarala, Frauke Engel, Anja Hartmann, Nicolaus von Wirén, Mohammad-Reza Hajirezaei

**Affiliations:** 1 Leibniz Institute of Plant Genetics and Crop Plant Research (IPK), Gatersleben, Germany; 2 Kötterheinrich Hortensienkulturen, Research & Development & Breeding, Lengerich, Germany; 3 Royal Botanic Gardens, Kew, Surrey UK

**Keywords:** *Hydrangea*, Phyllodulcin, Metabolomics, Transcriptomics, Mass spectrometry, Natural product chemistry, Biochemistry, Biotechnology, Plant sciences

## Abstract

Among the various natural products found in Hydrangea species, the bioactive dihydroisocoumarin, phyllodulcin (PD) stands out as a non-caloric, high-intensity sweetener, which is up to 800 times sweeter than sucrose. Additionally, PD possesses medicinal properties and plays plant-specific roles in pathogen resistance and abiotic stress tolerance. However, the biosynthetic pathway of PD in *Hydrangea* has remained unexplored. To identify intermediates and candidate genes involved in the biosynthesis of PD, we first conducted a screening approach, in which 14 out of 182 different *Hydrangea* accessions were selected for their contrasting foliar concentrations of PD and closely related hydrangenol (HD). Analysis of phenylpropanoid pathway (PPP) metabolites showed that accessions of *H. macrophylla* with high PD levels displayed distinct metabolite profiles compared to those with low PD concentrations. Specifically, caffeic acid, ferulic acid, and their derivatives, such as scopolin, scopoletin, esculetin, and fraxetin, were predominant in accessions with low PD concentrations. Conversely, the metabolite levels of phenylalanine, umbelliferone, p-coumaric acid, naringenin, resveratrol, and thunbergiol C (Thn C) were higher in accessions producing more PD. Transcriptome analysis revealed differentially expressed genes involved in phenylpropanoid biosynthesis, flavonoid biosynthesis, and stilbene biosynthesis pathways that are crucial for PD biosynthesis. Moreover, the identification of cyclase and ketoreductase genes, which were upregulated in accessions with high PD, provided further evidence for the biosynthetic pathway leading to PD. Based on metabolite profiling and gene expression data, we propose a hypothetical biosynthetic pathway for PD.

**Supplementary Information:**

The online version contains supplementary material available at 10.1038/s41598-026-47892-x.

## Introduction

Highly variable environmental conditions have led to the evolution of diverse metabolic pathways in plants that enabled them to respond to diverse stimuli and establish sophisticated relationships with co-evolving species through the production of biomolecules with specialized functions^1–3^. These secondary metabolites include an array of more than 200,000 diverse chemical compounds derived from multiple biosynthetic pathways^4^. Notably, the origin of most of these biomolecules is attributed to phenylpropanoid biosynthesis where a group of organic compounds derived from the amino acid L-phenylalanine go through a deamination process facilitated by L-phenylalanine ammonia lyase (PAL)^3,5^. Many of these PPP-derived molecules are bioactive and have not only stress-protective functions in plants but also therapeutic to humans^6^. In the plant *Hydrangea macrophylla,* PPP gives rise to a species-specific 3,4-dihydroisocoumarin called R-( +)- phyllodulcin (PD) which is 400–800 times sweeter than sucrose^7–9^. In the leaves, PD is naturally present in the form of phyllodulcin-β-D-glucoside. When the plant experiences various stresses, such as drought, wounding or senescence, native glucosidases within the plant hydrolyze these glucosides, converting PD to its aglycone form, which has a pleasantly sweet, minty taste^10^. This thermal hydrolysis is exploited to brew sweet tea in Japan from the leaves of the plant using hot water^8^. PD has been shown to have several beneficial properties in both traditional and modern medicine, including antibacterial, antimalarial, antifungal, antiulcer and anti-inflammatory effects^11,12^.

Although being a natural sweetener with documented bioactivity in traditional and modern medicine, a complete elucidation of PD biosynthetic pathway in *H. macrophylla* is still under progress. Early investigations using labeled ^14^C compounds indicated that the initiation of PD biosynthesis occurs through L-phenylalanine and trans-cinnamic acid^13–16^. These studies also suggested that branching from p-coumaric acid was a possible route for PD biosynthesis and hydrangenol (HD) could serve as a precursor in this pathway. The subsequent discovery of two key enzymes, p-coumaroyltriacetic acid synthase (CTAS) that catalyzes three decarboxylative condensations of p-coumaroyl-CoA and malonyl-CoA to yield p-coumaroyltriacetic acid (CTA) and its lactone (CTAL) and stilbenecarboxylate synthase (STCS) which converts dihydro-p-coumaric acid to stilbene carboxylates, such as 5-hydroxy-lunularic acid along with the availability of *H. macrophylla* genome greatly improved the understanding of phenylpropanoid metabolism in the plant providing insights of PD biosynthesis^17–19^. In parallel, thunberginols synthesized from resveratrol have also been proposed to be involved in PD biosynthesis^20,21^. While these studies lay the foundation for deciphering the PD biosynthetic pathway in *Hydrangea*, a comprehensive understanding would require further research into the genes, enzymes, and potential intermediates involved in this pathway. The current study was undertaken to elucidate PD and HD biosynthesis and metabolism in *Hydrangea* plants, using comparative transcriptomics and metabolomic profiling of PPP intermediates in a set of *H. macrophylla* accessions that were identified to differentially accumulate PD. Subsequently, downstream metabolomic analysis along with weighted gene co-expression network analysis (WGCNA) was employed to explore metabolite–gene relationships and progressively reconstruct the biosynthetic pathway.

## Results

### PD concentrations in fresh and dried leaves of selected *H. macrophylla* accessions

**Fig. 1:**
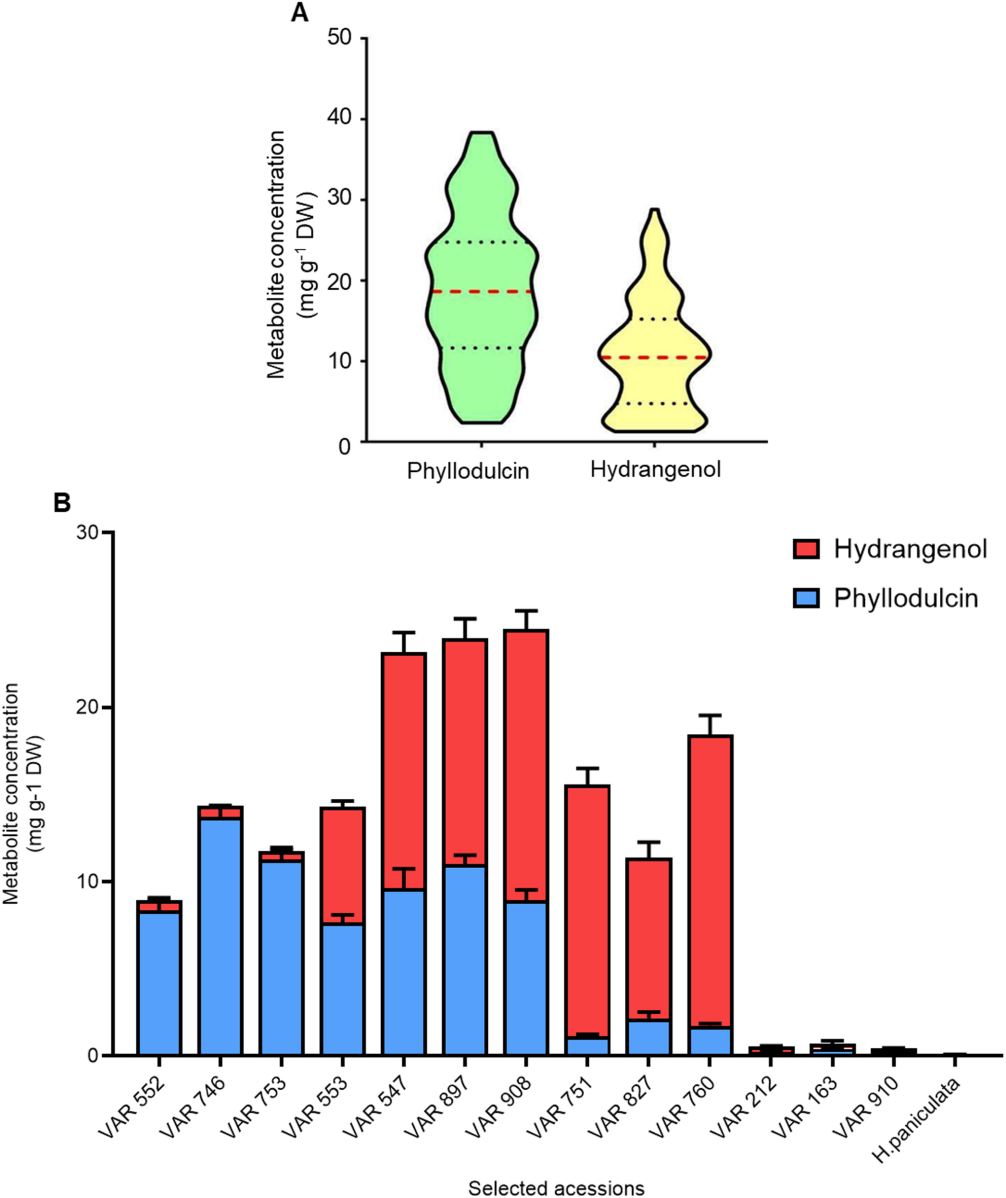
Screening and selection of 182 different Hydrangea accessions based on levels of phyllodulcin (PD) and hydrangenol (HD). (**A**) Biochemical variation of PD and HD concentrations in 182 H. macrophylla accessions. Violin plots shows the median by red dotted line, while the 3rd and 1st quartiles are represented by black dotted lines. (**B**) PD and HD concentrations in selected H. macrophylla accessions. Blue bars show the concentration of phyllodulcin while red bars show the concentration of HD. H. paniculata which does not produce PD or HD served as negative control. Bars represent means + SE (n = 6).

PD and HD concentrations of the 182 *H. macrophylla* accessions displayed wide variation aiding in the selection of different biological groups essential for the study. PD concentrations measured in the leaves of 75-day-old plants ranged from 1.36 to 38.34 mg g^−1^ DW, while HD concentrations ranged from 1.28 to 28.80 mg g^−1^ DW (Fig. 1A). Based on these measurements, the selected 13 accessions were assigned to four different groups according to their PD and HD concentrations and availability (Fig. 1B): high PD (VAR-552, VAR 746 and VAR-753), high PD and HD (VAR-553, VAR-547, VAR-897 and VAR-908), high HD (VAR-751, VAR-827 and VAR-760) and low PD and HD (VAR-212, VAR-910 and VAR-163).

A comparison of levels in freshly harvested and dried leaves revealed that the dried leaves of the selected lines contained up to eightfold higher levels of PD and HD (Fig. S1A, B) compared to their fresh counterparts (Fig. S1C, D). As expected, neither PD nor HD was detected in *H. paniculata* (Fig. S1A-D). Notably, apart from PD and HD other associated phenylpropanoids relevant to this study could not be detected in appreciable amounts from dried material.

### Variations in phenylpropanoid metabolism in *Hydrangea* accessions

**Fig. 2:**
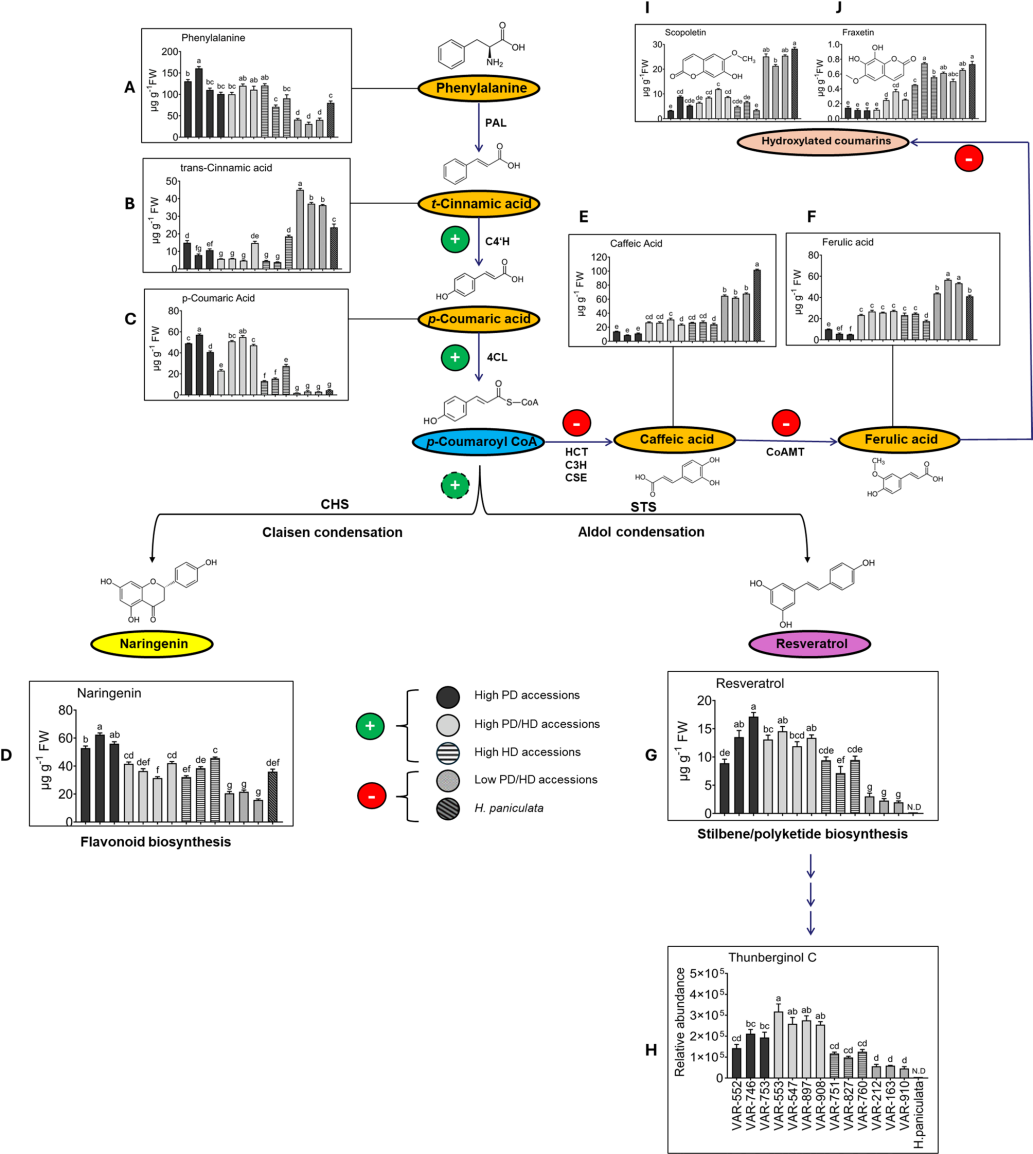
Levels of specific phenylpropanoids, determined in different *Hydrangea* accessions. Concentrations of (**A**) phenylalanine, (**B**) trans-cinnamic acid, (**C**) p-coumaric acid, (**D**) naringenin, (**E**) caffeic acid, (**F**) ferulic acid, (**G**) resveratrol, (**H**) thunberginol C, (**I**) scopoletin and (**J**) fraxetin measured in 14 selected *Hydrangea* accessions. Green and red circles represent accessions with high PD or HD and low to no PD or HD, respectively. Analysis was performed on freshly harvested, fully expanded, young upper leaves. Bars represent the means of 6 independent biological replicates (n = 6) and standard error. Dark bars show accessions with high PD, light grey bars accessions with high PD/HD, hatched bars accessions with high HD, dark grey bars accessions with low PD/HD and dark hatched bars *H. paniculata* as negative control. Different letters indicate significant differences among accessions according to one-way ANOVA followed by post-hoc Tukey’s test (*p* < 0.05). N.D., not detectable.

To investigate changes in phenylpropanoid metabolism in selected *H. macrophylla* accessions, the concentrations of 14 closely related phenylpropanoids were analyzed in freshly harvested leaves, as these metabolite levels are to be correlated with transcriptome data obtained from the same tissues. Relative to the low HD and PD lines, the high PD, high HD and high PD and HD lines showed significantly higher concentrations of phenylalanine, which serves as a starting compound in phenylpropanoid metabolism (Fig. 2A). Furthermore, all low PD and HD accessions and *H. paniculata* accumulated significantly higher concentrations of trans-cinnamic acid compared to the other groups (Fig. 2B). In plants, activated p-coumaric acid is branched to various metabolites, including umbelliferone, caffeic acid, naringenin, chalcone and resveratrol^22–24^. Plants with high PD and/or HD concentrations contained significantly higher levels of p-coumaric acid and naringenin compared to low PD and HD accessions and *H. paniculata* (Fig. 2C, D). The concentrations of scopoletin and fraxetin were higher in low PD and HD accessions and *H. paniculata* compared to high PD accessions (Fig. 2I, J). The levels of caffeic acid and ferulic acid were significantly higher in low PD and HD accessions compared to all other accessions (Fig. 2E, F). The levels of stilbenoids, especially resveratrol, were lower in the low PD and HD accessions compared to all other plants and were not detected in *H. paniculata* (Fig. 2G). Among the five different thunberginols, Thn C showed clear differences between different *Hydrangea* accessions. The relative abundance of Thn C was lower in low PD and HD accessions than in all other plants and Thn C was not detected in *H. paniculata* (Fig. 2H). These changes observed in the metabolite profiles of different *H. macrophylla* accessions reveal that plants with high PD, HD, or PD and HD concentrations accumulated naringenin and stilbenoids like resveratrol and Thn C, indicating that the preferred route for PD synthesis is likely through these metabolites.

**Fig. 3:**
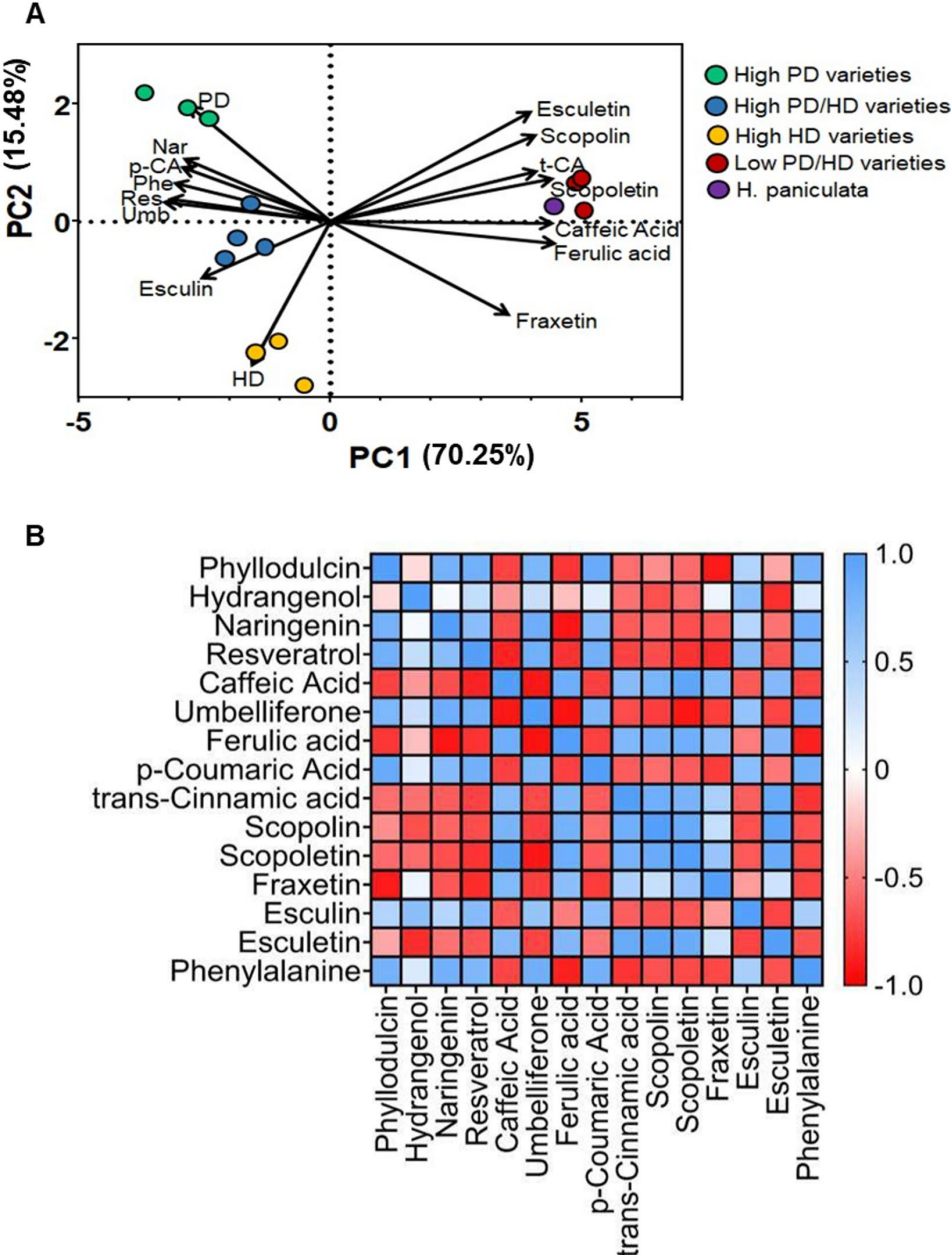
Multivariate analysis and metabolite interrelationships in *H.macrophylla* (**A**) PCA biplot for metabolite concentrations of different Hydrangea accessions with eigenvectors for individual metabolites. Each symbol represents an individual Hydrangea accession while arrows represent eigenvectors of 15 metabolites. The abbreviations Nar, p-CA, Phe, Res, Umb and t-CA represent naringenin, p-coumaric acid, phenylalanine, resveratrol, umbelliferone and trans-cinnamic acid, respectively. (**B**) Correlation analysis between phenylpropanoids in Hydrangea accessions. Pearson’s correlation analysis was performed among 15 metabolite concentrations of different Hydrangea accessions. Blue color represents high positive correlation (r = 1) and red color represents high negative correlation (r =  − 1). Metabolite analysis was performed on freshly harvested fully expanded upper leaves (n = 6).

Principal component analysis (PCA) was conducted using the metabolite concentrations of each accession to cluster accessions exhibiting similar metabolite trends. Principal component 1 (PC1) captured 70.25% of the variance in metabolite concentrations among accessions while principal component 2 (PC2) captured 15.48% of the variance. The PCA plot showed that each of the four-groups of lines formed distinct clusters, confirming that metabolite concentrations were effective to distinguish these groups (Fig. 3A). In the loading plot, the vectors differentiating high PD or high HD were almost orthogonal, indicating that the analyzed metabolites were decisive for separation of the accessions. Accessions VAR-552, VAR-746, and VAR-753 clustered along the PD vector projection, implying that PD could be a key factor in this grouping (Fig. 3A). Similarly, VAR-751, VAR-760 and VAR-827 clustered closer to the HD eigenvector projection, suggesting that HD may be responsible for this clustering. Accessions VAR-212, VAR-163, VAR-910 and *H. paniculata* clustered close to the trans-cinnamic acid, scopolin and caffeic acid eigenvectors. On the other hand, VAR-553, VAR-908, VAR-897 and VAR-547 clustered closer to the eigenvectors of umbelliferone, resveratrol and phenylalanine (Fig. 3A).

Furthermore, analysis of the PPP intermediates showed strong positive correlations between PD concentrations and the concentrations of naringenin, resveratrol, umbelliferone, p-coumaric acid, and phenylalanine (Fig. 3B). Conversely, there were strong negative correlations between PD and caffeic acid, ferulic acid, trans-cinnamic acid, scopolin, scopoletin, fraxetin and esculetin (Fig. 3B).

### Differential expression of genes involved in PPP and associated pathways

To compare the genetic regulation of the PPP in *Hydrangea* lines with similar or contrasting PD and HD levels, an RNA sequencing approach was conducted using leaf RNA of 12 accessions used for metabolote profiling. Notably, *H. paniculata*, which does not contain PD, exhibited a different expression profile, characterized by a strong down-regulation of PPP genes (Fig. S2A), in contrast to other accessions with high or significant PD levels. In all other accessions, distinct clusters of up-regulated and down-regulated genes were identified, regardless of the PD concentration (Fig. S2A).

Next, differentially expressed genes were subjected to Gene Ontology (GO) analysis, which resulted in distinct gene expression profiles in *Hydrangea* accessions containing high PD or high HD (Fig. S2B). Among the biological processes, metabolic and cellular processes were highly represented. In the molecular function category, genes related to DNA binding, protein binding or histone binding and catalytic activity, were predominant (Fig. S2B).

**Fig. 4:**
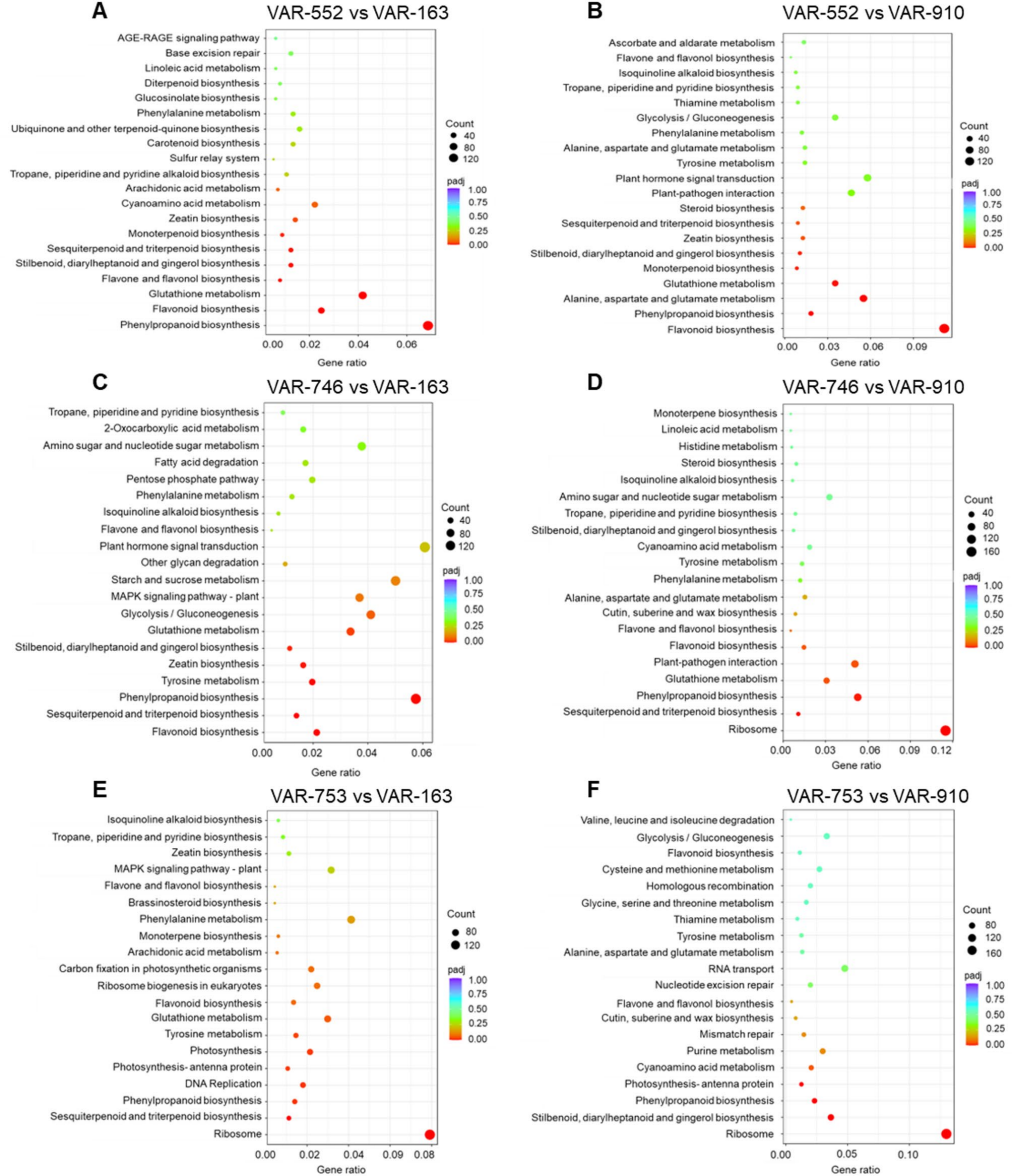
The assignment of genes to different pathways in high PD (VAR-552, VAR-746, VAR-753) and low PD (VAR-163, VAR-910) containing accessions of *H. macrophylla*. (**A**-**F**) Dot plot of the KEGG enrichment analysis showing the gene ratio (the percentage of total DEGs) assigned to the top 20 pathways in the study group. The dot size represents the number of genes and the color of the dot is based on the p-value adjusted to the sample distribution (padj value) and indicates the significance of pathway enrichment. KEGG annotates genes at the pathway level^25,26^.

Furthermore, KEGG (Kyoto Encyclopedia of Genes and Genomes) enrichment analysis was performed to assign differentially expressed genes to biochemical pathways. When comparing the high PD and HD or high HD accessions with the very low PD accessions, pathways related to phenylpropanoid biosynthesis, ribosome, flavonoid biosynthesis, flavone and flavonol biosynthesis, or stilbenoid, diarylheptanoid and gingerol biosynthesis were among the top 20 highly expressed metabolic pathways^25,26^ (Fig. 4). Conversely, these pathways did not show significant enrichment when comparing the transcriptome profiles of accessions with PD and/or HD to the negative control *H. paniculata* (Fig. S3).

These results indicate that differentially expressed genes associated with flavonoid biosynthesis, phenylpropanoid biosynthesis and stilbene biosynthesis were highly enriched in high PD and/or HD lines when comparing the transcriptome with that of low PD and/or HD lines.

### Metabolite-gene relationship in PPP and identification of contrasting gene expressions between high and low PD accessions

**Fig. 5:**
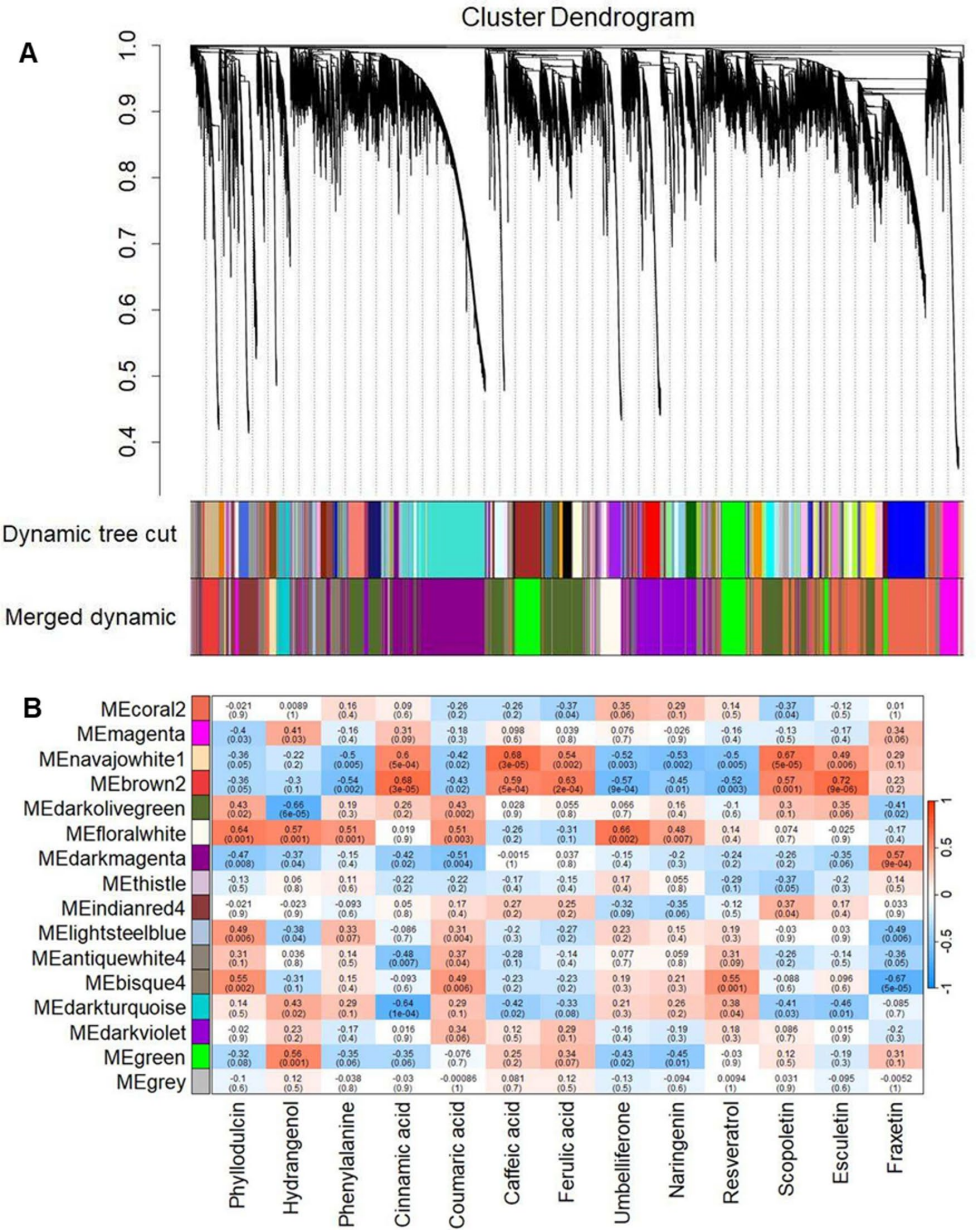
Correlation analysis between the identified genes and metabolites using Weighted Gene Co-expression Network Analysis (WGCNA). (**A**) Hierarchical clustering dendrogram of the genes. Gene clustering tree (dendrogram) obtained by hierarchical clustering of adjacency-based dissimilarity to detect 85 co-expression clusters, with corresponding color assignments shown as a dynamic tree section. The closeness of the branches indicates the similarity between gene sets, and genes with similar expression patterns are grouped together in the same module. (**B**) Modules with strongly correlated eigengenes were merged based on threshold to assign highly co-expressed genes into 16 separate modules. Color bars reflect module assignments before and after the merging of closed modules. Each color represents a module and the gray module indicates none co-expression among the genes. Analysis was done on 22,980 genes obtained from RNA sequencing of 11 *H. macrophylla* accessions.

To investigate the relationship between genes involved in the PPP and metabolite concentrations, a weighted gene correlation network analysis (WGCNA) was performed, followed by correlation of eigengenes with metabolite levels. To enhance validity of the network, genes that were not expressed in all samples were eliminated. Subsequently, high-quality genes with a variance cut-off greater than 0.55 were selected, resulting in a total of 22,980 genes, which exhibited significant variance among accessions. A gene cluster dendrogram was constructed, with each branch representing a gene cluster with highly correlated expression levels (Fig. 5A). In total, 85 modules marked by different colors were obtained, each containing co-expressing genes. Based on the module eigengene similarity, 16 expression modules were observed, with the dark olive-green module containing most genes (5065) and the grey module containing the least (68) (Fig. 5B). Pearson’s correlations were performed to estimate module-metabolite relationships.

Differential expressions of the genes in the floralwhite, bisque4, lightsteelblue and darkolivegreen modules showed a high positive correlation with PD concentration, while the floralwhite, darkturquoise, green and magenta modules correlated with HD concentrations. Phenylalanine concentration positively correlated with the lightsteelblue and floralwhite modules, while p-coumaric acid concentration positively correlated with darkturquoise, floralwhite, lightsteelblue, antiquewhite4 and bisque4. The navajowhite1 and brown2 modules showed high positive correlation with trans-cinnamic acid, caffeic acid, ferulic acid and esculetin concentrations, whereas umbelliferone and naringenin concentrations correlated positively with the floralwhite module. Resveratrol concentration correlated positively with bisque4 and darkturquoise and scopoletin concentration correlated positively with navajowhite1, brown2 and indianred4. Fraxetin concentration correlated positively with the dark magenta module. Notably, the floralwhite module correlated positively with PD, HD, phenylalanine, p-coumaric acid, umbelliferone and naringenin, suggesting a common regulatory network for these metabolites.

**Fig. 6:**
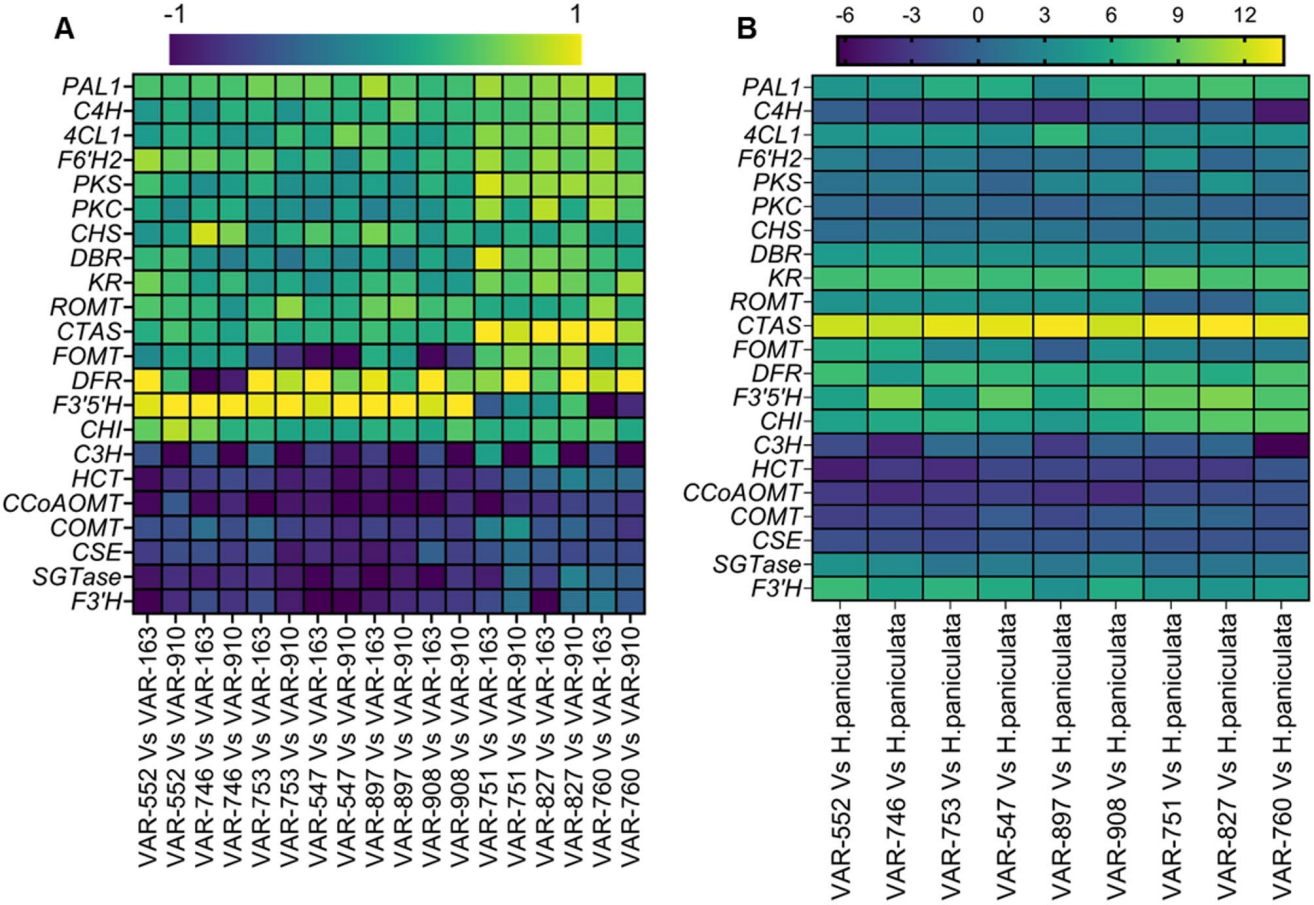
Expression patterns of module-specific genes involved in the PPP and associated pathways in Hydrangea accessions. (**A**) Heatmap of selected genes involved in PPP, flavonoid biosynthetic pathway and stilbene biosynthetic pathway among H. macrophylla accessions. The comparison was between a high PD and/or HD accession with low PD and HD accessions (VAR-163 and VAR-910). (**B**) Heatmap of selected genes involved in PPP, flavonoid biosynthetic pathway and stilbene biosynthetic pathway while comparing H. macrophylla and H. paniculata. Differentially expressed genes were selected based on module-trait relationships derived from WGCNA, where each gene was present in a module, which correlated to individual metabolite concentrations (padj < 0.05). Each square block represents the normalized log2-foldchange values of specific genes in each study group. Yellow represents the highest relative expression level of the gene and dark violet represents the lowest relative expression level of the gene. Heatmaps with associated statistical analysis were generated using GraphPad Prism version 10.0.0 for Windows, GraphPad Software, Boston, Massachusetts USA, www.graphpad.com.

By searching for genes in modules highly correlated with metabolite concentrations and related to the metabolic pathways from KEGG enrichment analysis (flavonoid biosynthesis, phenylpropanoid biosynthesis and stilbene biosynthesis), 22 genes were identified and displayed in Table 1. Among these, the genes involved in stilbene biosynthesis and associated pathways, such as p-coumaroyltriacetic acid synthase (*CTAS*), resveratrol di-O-methyltransferase (*ROMT*), keto reductase (*KR*), type III polyketide synthase (*PKS*) and polyketide cyclase (*PKC*), were upregulated in high PD and/or HD accessions (Fig. 6). These genes were of particular interest due to the ability of the corresponding enzymes to catalytically produce structurally similar dihydroisocoumarins to those of PD and HD.

**Table 1:** Correlation analysis between identified genes and metabolites using WGCNA. PD: phyllodulcin, HD: hydrangenol. For gene names refer to the manuscript.

Sl no	Module color	Gene count	Gene names Up-regulated	Gene names Down-regulated	Positive correlation with metabolites
1	darkolivegreen	5065	*PAL1*, *4CL1*, *F6’H*1, *PKS*, *PKC*	*F3’H*	PD, p-coumaric acid, trans-cinnamic acid
2	coral2	4153			
3	darkmagenta	3294			
4	darkviolet	2508		*COMT*	
5	Green	1907			HD
6	bisque4	1656	*CTAS*, *ROMT*, *CHS*, *CHI*		PD, p-coumaric acid, resveratrol
7	indianred4	923			scopoletin
8	floralwhite	906	*4CL1*, *F6’H2*, *DFR*, *DBR*		PD, HD, phenylalanine, p-coumaric acid, umbelliferone, naringenin
9	Magenta	768			HD
10	brown2	553		*HCT*, *C3H*, *CCoAOMT*, *SGT*	Caffeic acid, ferrulic acid, scopoletin
11	darkturquoise	432			HD, resveratrol
12	navajowhite1	379	*F6’H2*	*CSE*	trans-cinnamic acid, caffeic acid, ferulic acid, esculetin, scopoletin
13	lightsteelblue	172	*C4H*		PD, phenylalanine, p-coumaric acid
14	Antiquewhite4	123			p-coumaric acid
15	Thistle	73			
16	Grey	68			
	Total	22,980

### Validation of the expression of candidate genes by quantitative real-time PCR (qRT-PCR)

The genes identified by RNA sequencing were verified by quantitative real-time PCR. For this purpose, specific primers were designed from the sequences of 10 candidate genes involved in phenylpropanoid, flavonoid and stilbenoid biosynthesis, which showed different expression levels in all experimental groups (Table S3). The selected genes were *DFR, DBR, CTAS, PKC, 4CL, PAL1, KR, HCT, CCoAOMT* and *COMT*. Gene expression levels were expressed as fold-change and log2-transformed by comparing the same study groups as in the RNA sequencing experiment. Examination of gene expression levels by qRT-PCR revealed that transcript abundance of *DBR, CTAS, PKC, 4CL, PAL1* and *KR* was higher in high PD and/or HD accessions than in low PD and HD accessions (Figs. S4, S5). Similarly, the genes *HCT, CCoAOMT* and *COMT*, which are involved in caffeic acid synthesis and related downstream metabolites, were found to be downregulated when comparing the same set of accessions (Fig. S4, S5). These results confirmed the associations between gene expression patterns and metabolic pathways leading to PD biosynthesis.

## Discussion

### Genetic regulation of PD and HD biosynthesis in *H. macrophylla*

The current study which used comparative transcriptome analysis and KEGG pathway analysis in accessions with high PD and/or HD levels or with low PD and HD levels revealed significant enrichment of genes belonging to PPP and its sub pathways: flavonoid biosynthesis and stilbene biosynthesis (Fig. 4). Large differences in gene expression between *H. macrophylla* accessions and *H. paniculata* were observed by hierarchical clustering (Fig. S3). The expression levels of *HCT* (p-coumaroyl CoA → p-coumaroyl shikimate; caffeoyl shikimate → caffeoyl CoA)^27,28^, *C3H* (p-coumaroyl shikimate → caffeoyl shikimate)^29,30^, *CCoAOMT* (caffeoyl CoA → feruloyl CoA)^28,30^, *CSE* (caffeoyl shikimate → caffeate) , *COMT* (caffeate → ferulate) and *SGT* (scopoletin → scopolin) were found to be downregulated in accessions with high PD and/or HD levels compared to those with low PD and HD levels (Fig. 6). This suggests that in low PD and HD accessions, there is a higher abundance of genes and metabolites associated with caffeic and ferulic acid, which are part of the alternative pathway leading away from PD and HD biosynthesis^16^. Furthermore, genes encoding the enzymes CHS (p-coumaroyl CoA → naringenin chalcone), CHI (naringenin chalcone → naringenin), F3′5’H (dihydrokaempferol → dihydromyricetin), DFR (dihydromyricetin → leucodelphinidin), ROMT (resveratrol → pinostilbene → pterostilbene) and FOMT showed upregulation in high PD and/or HD accessions and downregulation in low PD and HD accessions (Fig. 6) indicating that these genes might play an important role in the biosynthesis of phyllodulcin.

**Fig. 7:**
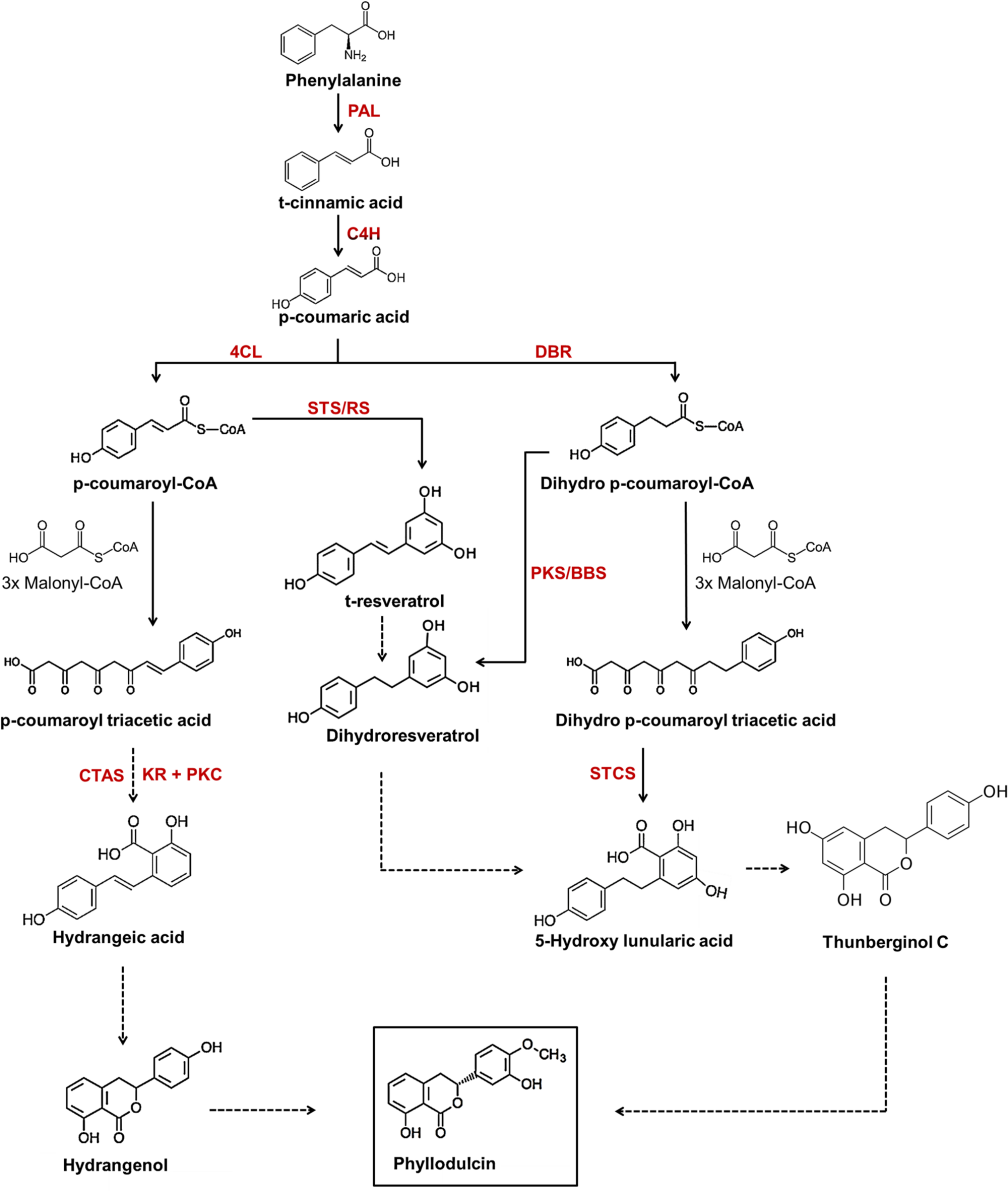
Proposed scheme of phyllodulcin biosynthesis. Phenylalanine is converted by phenylalanine ammonia lyase (PAL), cinnamate 4-hydroxylase (C4H) and 4-coumarate-CoA ligase (4CL) to p-coumaric acid. p-coumaric acid is either converted to p-coumaroyl-CoA and p-coumaroyltriacetic acid and finally to hydrangenol, which may serve as a precursor for phyllodulcin formation. p-coumaric acid can also be converted to dihydro-p-coumaroyl-CoA and further to dihydro-p-coumaroyl triacetic acid and hydroxylunularic acid or dihydroresveratrol. Alternatively, p-coumaroyl-CoA can be converted to resveratrol and dihydroresveratrol. The latter and hydroxyunularic acid can be converted to thunberginol C, which is most likely the direct precursor for the synthesis of phyllodulcin. 4CL, 4-coumarate-CoA ligase; STS/RS, stilbene synthases/resveratrol synthase; DBR, double bond reductase; PKS, type III polyketide synthase; BBS, bibenzyl synthase; KR, ketoreductase; PKC, polyketide cyclase; CTAS, p-coumaroyltriacetic acid synthase; STCS, stilbene carboxylate synthase. Enzymes in each step are represented in red fonts and metabolites are represented in black fonts. The solid lines and dashed lines represent reported and proposed steps in the pathway respectively.

By integrating the metabolite and transcriptome data as performed by WGCNA (Fig. 5), accessions with high PD and/or HD levels exhibited enhanced flavonoid biosynthesis (involving metabolites derived from naringenin chalcone) and stilbene biosynthesis (involving metabolites derived from resveratrol) compared to low PD/HD accessions. In our comparative metabolite analysis, a positive correlation was found between the concentrations of PD, HD and phenylalanine, confirming the essential role of phenylalanine in PD biosynthesis, with accessions possessing higher phenylalanine producing more PD (Fig. 3A, B). Accessions with high levels of PD and/or HD also showed increased levels of p-coumaric acid (Fig. 3B), and these levels correlated positively with those of PD and HD (Fig. 3A). Moreover, the higher expression levels of *4CL* in high PD and/or HD accessions compared to low PD/HD accessions can be attributed to the activation of p-coumaric acid to p-coumaroyl CoA, which appeared in our approach as a critical intermediate in the pathway leading to PD (Fig. 7). Conversely, a negative correlation was observed between PD and HD concentrations and caffeic acid, ferulic acid, scopolin, scopoletin and esculetin, while no correlation was found for fraxetin (Fig. 3A, B). This suggests that these metabolites may have limited or no role as upstream intermediates in PD and HD biosynthesis. It is also noteworthy that the concentration of these coumarins (except fraxetin) were generally low in accessions with high PD and/or HD levels (Fig. 3A, B).

In contrast to the mentioned coumarins, the metabolites umbelliferone, naringenin, and resveratrol displayed a positive correlation with PD and HD concentrations (Fig. 3A, B), suggesting that these metabolites are associated with PD and HD biosynthesis. Previous studies have identified a wide variety of dihydroisocoumarin-related metabolites in *Hydrangea*, including thunberginols C, D, E, and G^31,32^. However, the connections between these metabolites and PD biosynthesis have not been studied by integrating metabolomics and transcriptomics. In this study, accessions characterized by high PD and/or HD levels exhibited elevated concentrations of these metabolites, along with Thn C (Fig. 2H). p-coumaroyltriacetic acid synthase (CTAS) plays a key role in the conversion of p-coumaroyl CoA to p-coumaroyltriacetic acid lactone (CTAL). Previous studies have hypothesized that CTAS, in conjunction with a cyclase and a ketoreductase, can facilitate the synthesis of hydrangeic acid. Subsequently, hydrangeic acid may undergo further modifications within the plant, ultimately leading to the formation of HD^17–19^. In the current research, significantly higher transcript levels of *CTAS* were observed in the accessions characterized by high concentrations of PD and/or HD, with the highest expression detected in the accessions containing both PD and HD (Fig. 6A). Using quantitative RT-PCR, the present study revealed up-regulation of type-III polyketide synthases (*PKS*), ketoreductase (*KR*), polyketide cyclase (*PKC*) and double-bond reductase (*DBR*) in accessions with high PD and/or HD (Figs. S4, S5). Interestingly, *CTAS* was absent in *H. paniculata,* and *PKC* together with *KR* exhibited elevated expression levels when all *H. macrophylla* accessions were compared with *H. paniculata*, where PD and HD are not detected (Fig. 6A). This observation suggests that these genes probably play a central role in the biosynthesis of PD and HD. The abundance of these genes, particularly *KR* and *PKC*, could potentially form a multi-enzyme complex responsible for the synthesis of stilbene carboxylic acid (hydrangeic acid), in line with a hypothesis raised in previous researches^17–19^. A similar set of genes involving type-*III PKS, KR* and *PKC* has been proposed to be responsible for the production of lunularic acid, another stilbene carboxylic acid, in *Cannabis sativa*^33^. Thus, several alternative pathways for the biosynthesis of PD and HD have been proposed in the literature, and an intriguing hypothesis revolves around the role of thunberginols in this biosynthetic pathway. Thunberginols and HD have been identified as catabolic products in the faeces of PD-fed rats^34,35^. Furthermore, a proposed biosynthetic pathway starting from resveratrol to dihydroresveratrol, 5-hydroxy-lunularic acid, and Thn C has been proposed^20^. In addition, a type-III polyketide synthase (*PKS*) responsible for the conversion of dihydro-paracoumaroyl-CoA to dihydroresveratrol has been reported in *Cannabis sativa*^36^. In the current study, higher concentrations of resveratrol were observed in accessions with high PD and/or HD levels compared to those with low PD/HD levels (Fig. 2G). The enzyme responsible for the downstream processing of resveratrol, ROMT, also showed increased expression levels in accessions with high PD and/or HD compared to low PD/HD accessions (Fig. 6A). At the same time, a higher relative abundance of Thn C was found in the accessions with high PD and/or HD levels compared to those with low PD and HD levels (Fig. 2H). However, it is noteworthy that resveratrol and Thn C were not detected in the *H. paniculata* accession (Fig. 2H) supporting that resveratrol and Thn C might be involved in the synthesis of phyllodulcin. Unfortunately, 5-hydroxy-lunularic acid, a metabolite involved in the same pathway, could not be evaluated due to the unavailability of a reference standard at the time of the study and the challenges associated with proper chromatographic separation. However, the presence of 5-hydroxylunularic acid and its closely related metabolites in *Hydrangea* has been very well reported^37–39^.

### Proposed model for phyllodulcin biosynthesis

Based on the findings from the metabolome and transcriptome data in the present research, coupled with an extensive review of the relevant literature on the subject, a pathway outlining the biosynthesis of PD has been devised (Fig. 7). In low PD and HD-containing *H. macrophylla* accessions, there is a substantial diversion of metabolic flow away from dihydroisocoumarin biosynthesis. Conversely, in accessions containing high PD, HD or PD and HD levels, the pathway is directed towards dihydroisocoumarin biosynthesis. This clear differentiation between accessions based on their biochemical concentrations is evident in the PCA biplot (Fig. 3A) and pathway description in Fig. 2. In particular, naringenin, phenylalanine, resveratrol, umbelliferone are responsible for the differences observed in the high PD accessions. Similarly, esculetin, scopolin, trans-cinnamic acid, scopoletin, caffeic acid and ferulic acid are most characteristic for the low PD/HD accessions (Fig. 2). These pathway shifts, coupled with variations in the expression of key genes within the PPP, clearly show that the initial reactions for PD biosynthesis are the same as for many other secondary metabolites, but downstream of p-coumaric acid, representing a branching point, three distinct pathways are likely to lead to PD biosynthesis: one going through HD, another through resveratrol and a third through Thn C (Fig. 7). Further detailed elucidation of the PD biosynthesis pathway needs to be explored by identifying the involved genes, gene products and associated chemical reactions, in the best case together with a reconstitution of the pathway in a non-PD containing plant species, such as tobacco, by using transgenic approaches and/or feeding experiments with labeled precursors.

## Materials and methods

### Plant material and sample processing

Fresh, fully expanded young upper leaves from 182 *Hydrangea macrophylla* accessions were obtained from Koetterheinrich Hortensienkulturen (Lengerich, Germany) and subsequently screened for PD and HD concentrations after drying the leaf tissue. Thirteen accessions were selected and chosen for metabolomic and transcriptomic experiments (Table S1). The selected 13 accessions namely, VAR-552, VAR 746, VAR-753, VAR-553, VAR-547, VAR-897, VAR-908, VAR-751, VAR-827, VAR-760, VAR-212, VAR-910 and VAR-163, were grown from the cutting stage in the greenhouse at IPK-Gatersleben under controlled conditions, with an 18 h photoperiod, a light intensity of 200 μmol m^-2^ s^-1^, a temperature of 21/19 °C (day/night) and a relative humidity of 60%. The plants were randomized and grouped daily. The plants were allowed to grow for 75 days, at which point freshly expanded leaves were harvested, immediately frozen, ground to a fine powder and stored at -80 °C until use. These materials were used for metabolomic and transcriptomic analyses. Additionally, *Hydrangea paniculata*, a phylogenetically related species that naturally lacks both PD and HD^40^ (Fig. 1), was used as a negative control in the study. The contrast in metabolic and genetic makeup between these species made *H. paniculata* a good candidate for this study.

### Chemicals

LC–MS grade acetonitrile, methanol, and n-hexane used in this experiment were procured from Carl Roth (Karlsruhe, Germany). Formic acid was obtained from Thermo Fisher Scientific (Germany). Analytical standards essential for the quantitative measurements of PD and HD were provided by Symrise AG, (Dr. Ley, Holzminden, Germany). Additionally, the analytical standards required for phenylpropanoid analysis were purchased from Sigma Aldrich (Merck AG, Taufkirchen, Germany).

### Extraction of PD and HD

Both PD and HD were extracted with slight modifications to an existing method that included drying and fermentation steps, specifically using accelerated solvent extraction (ASE) as described previously^41,42^. Briefly, 5–10 mg of powdered tissue, both fresh and dried at 40 °C for 48 h, was fermented with 0.2 mL of ultrapure water for 2 h at 40 °C. Then, 1.8 mL of methanol was added, and the mixture was incubated in an ultrasonic bath for a further 2 h at 40 °C. The supernatant was separated by centrifugation at 13,000 RPM for 15 min and 1 mL of methanol was added to the sediment, which was then sonicated for 1 h. The supernatant from this fraction was combined with the previous fraction after centrifugation at 13,000 RPM for 15 min. The final mixture was collected and passed through Strata C18 columns (55 µm, 70 Å, 100 mg/ml, Phenomenex, Germany) preconditioned with 1 mL of methanol and eluted with 1 mL of methanol. The final volume was collected and subjected to LC–MS analysis.

### Extraction of phenolic compounds

For the extraction of phenolics, including p-coumaric acid, trans-cinnamic acid, caffeic acid, ferulic acid, naringenin and trans-resveratrol, a liquid extraction with methanol was performed with slight modifications based on the method described previously^43^. 5–10 mg of finely ground plant tissue was combined with 1 mL of 80% methanol and stirred in an ultrasonic bath for 1 h at 30 °C. The resulting extract was then centrifuged at 13,000 RPM for 15 min at 4 °C and the extraction process was repeated. The supernatant was then filtered through a 0.45 µm membrane filter and collected in new Eppendorf tubes for further analysis.

### Extraction of coumarins

Extraction of scopolin, scopoletin, esculetin, fraxetin and umbelliferone was performed with slight modifications based on a previously published method^44^. Briefly, 1 mL of methanol was added to the finely ground and powdered samples, which were then subjected to sonication for 1 h, followed by incubation in the dark at 4 °C for a further 2 h. All samples were then centrifuged at 13,000 RPM for 15 min, and the resulting supernatants were carefully transferred to new Eppendorf tubes. For further processing, the extracts were dried in a vacuum centrifuge for 2 h at 45 °C (Christ, RVC 2–33 RI, Germany). Subsequently, 100 μL of 80% methanol was added to the dried extracts to dissolve the compounds and incubated overnight at 4 °C. The next day, the extracts were vortexed for 10 min and separated into 50 μL aliquots. These samples were stored at -20 °C until analysis by LC–MS.

### UPLC-MS/MS analysis

UPLC-MS/MS analyses were performed on an Agilent 1290 UPLC system coupled to an Agilent 6490 triple quadrupole mass spectrometer. The chromatographic separation was performed on an ZORBAX RRHD Eclipse Plus C18, 95 Å, 2.1 × 50 mm, 1.8 µm column at a flow rate of 0.45 mL/min and a column temperature of 40 °C (Agilent Technologies, Waldbronn, Germany). The separation was performed with a gradient of solvent A (water) and B (acetonitrile), both containing 0.1% formic acid (v/v). The initial percentage of B was 10%, increased linearly to 80% in 5 min and then re-equilibrated to the original conditions for 6 min. ESI–MS/MS analysis was performed in positive and negative ionization mode using nitrogen as drying and nebulizing gas. The gas flow was set at 12.0 l/min at 250 °C and the nebulizer pressure was 30 psi. The capillary voltage was 2 kV and the residence time was 20. MassHunter optimizer software was used to select precursor ions using MS2 Selected Ion Monitoring (SIM), product ions using product ion scan for each precursor ion and optimum collision energy for each transition using multiple reaction monitoring (MRM) acquisition mode. Product ions were selected as the most abundant ions in a composite product ion scan.

spectrum obtained for a given precursor ion at multiple collision energies. Five different concentrations of selected 14 metabolites were used to prepare a calibration curve from a range of 0.01–50 µg per ml and the absolute quantification was performed using a single multiple reaction monitoring (MRM) transition for each analyte (Table S2). The limit of quantification (LOQ) for the coumarins was measured by triplicate injections of the standard solutions based on signal to noise ratio of 10. Agilent MassHunter software (B.07.01, Agilent Technologies, United States) was used for data acquisition as well as final qualitative and quantitative analysis.

### RNA isolation, sequencing and analysis

To isolate total RNA from the plant tissues, the Spectrum™ Plant Total RNA Kit, obtained from Sigma Aldrich, was employed following the manufacturer’s guidelines. The complete detailed procedure is described in Supplementary protocol S1.

### Validation of gene expression using qRT-PCR

The RevertAid First Strand cDNA Synthesis Kit from Thermo Scientific was utilized to perform cDNA synthesis, employing 0.3 µg of total RNA as starting material. The synthesis was initiated using oligo(dT) primers. The primers for the quantitative real-time polymerase chain reaction (qRT-PCR) were created through primer design software Primer3, and they were subsequently synthesized by the company Metabion (Germany). The list of primers designed for validation with qRT-PCR are represented in Table S3. The detailed procedure is described in Supplementary protocol S2.

### Weighted gene co-expression network analysis (WGCNA)

WGCNA is a data-driven method that discovers co-clustered gene sets (modules) based on weighted correlations among gene transcripts. This comprehensive method is used to analyze pairwise correlations between variables, often genes, in a dataset. It constructs a network where nodes represent variables and edges represent weighted correlations, allowing the identification of clusters of highly correlated variables, known as modules. These modules can then be related to internal and external traits, helping to uncover complex and functional relationships within the data. For the construction of gene co expression networks, weighted gene co-expression network analysis (WGCNA) was performed on 22,980 genes having high variance among the accessions, which were obtained from RNA sequencing of *Hydrangea* accessions. The WGCNA (version 1.72–1) R software package is a comprehensive collection of R functions for performing various aspects of weighted correlation network analysis. This R package included functions for network construction, module detection, gene selection, calculations of topological properties, data simulation, visualization, and interfacing with external software^45^.

### Statistical analysis

Statistical analysis was performed using GraphPad Prism 9.5.1 (GraphPad Software Inc.) and R (version 4.0.3). One-way ANOVA with post hoc Tukey’s test (p ≤ 0.05) was used for multiple comparisons. Paired sample t-tests were used to assess the significance of differences between the control and treatment groups.

## Supplementary Information

Supplementary Information.

## Data Availability

The data that supports the findings of this study are openly available within the article and in online Supporting Information files. The RNA sequencing data can be accessed through the European Nucleotide Archive under project code: PRJEB79406.
