# Supplementary material for: Elucidation and functional characterization of the biosynthetic pathway of the natural sweetener phyllodulcin in Hydrangea macrophylla
Source: Sci Rep. 2026 Apr 9;16:12044. doi: 10.1038/s41598-026-47892-x (PMC13068954; doi:10.1038/s41598-026-47892-x)
Supplement: Supplementary file 1 — Supplementary Information. [file 41598_2026_47892_MOESM1_ESM.docx]

**Supplementary Figures**


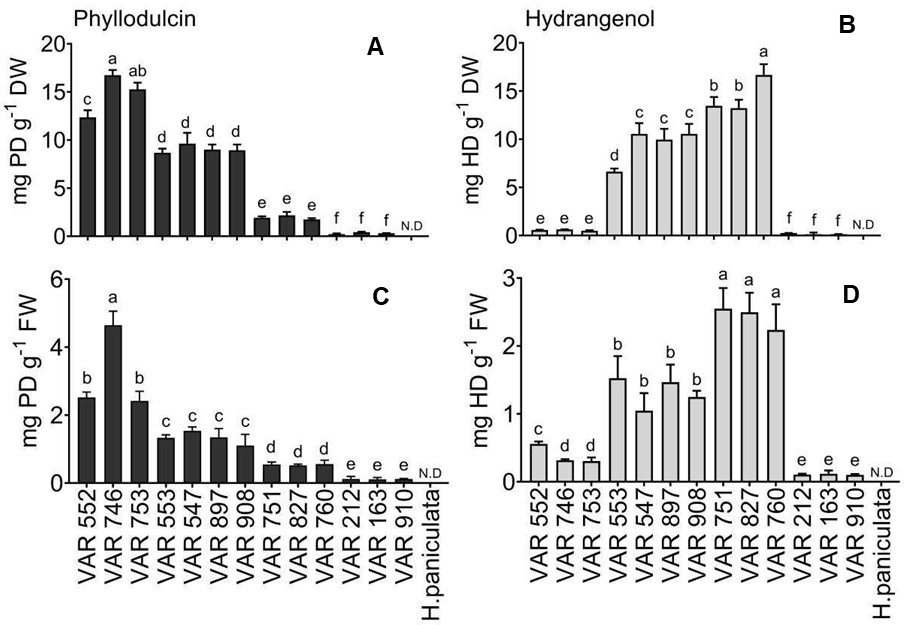


**Fig S1: Comparison between fresh and dried leaf tissues for phyllodulcin (PD) and hydrangenol (HD) concentrations in selected Hydrangea accessions.** (A) PD and (B) HD concentrations in dried leaves. (C) PD and (C) HD concentrations in freshly harvested leaves. Analysis was performed on fully expanded young upper leaves. Bars represent mean + SE (n=6). Not detected (N.D.) indicates the absence of the corresponding metabolite. Different letters indicate significant differences between accessions according to one-way ANOVA followed by post- hoc Tukey's test (p<0.05).


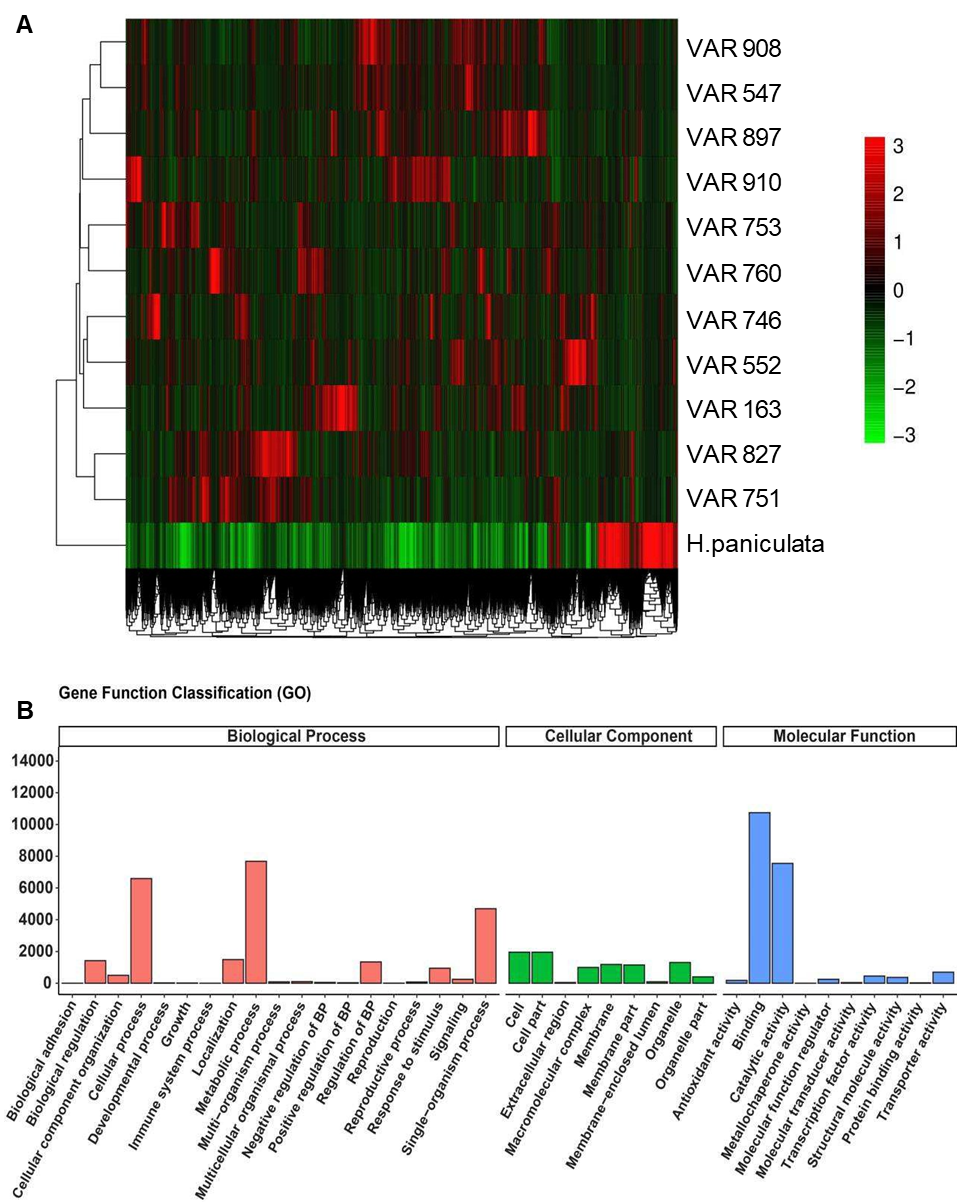


**Fig S2: Relative gene expression patterns among *Hydrangea* accessions.** (A) Heatmap showing the relative gene expression patterns in *H. paniculata* and 11 different accessions of H. macrophylla. Normalized expression data using fpkm values of genes were used to perform the up- and down regulated genes. Green colour represents downregulated genes and red colour represents upregulated genes (B) Gene Ontology (GO) analysis of differentially expressed genes classified by biological process, cellular component and molecular function. The 38 most significant terms were selected for display. The data were obtained from full length transcriptomes of leaf tissues of all Hydrangea accessions. Only GO terms with padj < 0.05 are shown. Heatmap generated in R version 4.4.0 using the pheatmap package version 1.0.13 (<https://cran.r-project.org/package=pheatmap>).


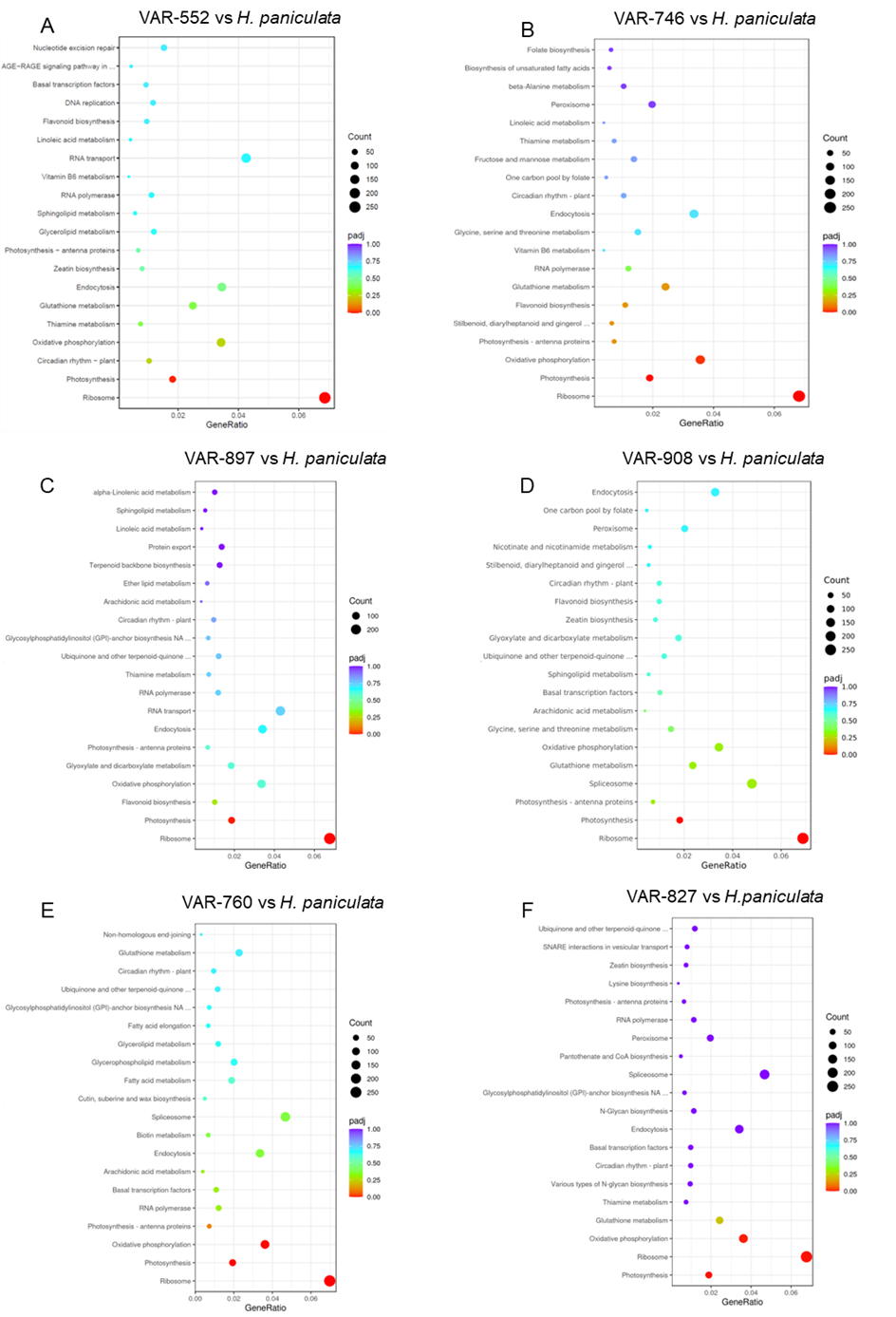


**Fig S3:** The assignment of genes to different pathways in high PD (A, B, VAR-552, VAR-746), high PD/HD (C, D, VAR-897, VAR-908) or high HD (E, F, VAR-760, VAR-827) and the negative control H. paniculata . (A-F) Dot plot of the KEGG enrichment analysis showing the gene ratio (the percentage of total DEGs) assigned to the top 20 pathways in the study group. The dot size represents the number of genes and the colour of the dot is based on the p-value adjusted to the sample distribution (padj value) and indicates the significance of pathway enrichment. KEGG annotates genes at the pathway level^25,26^.


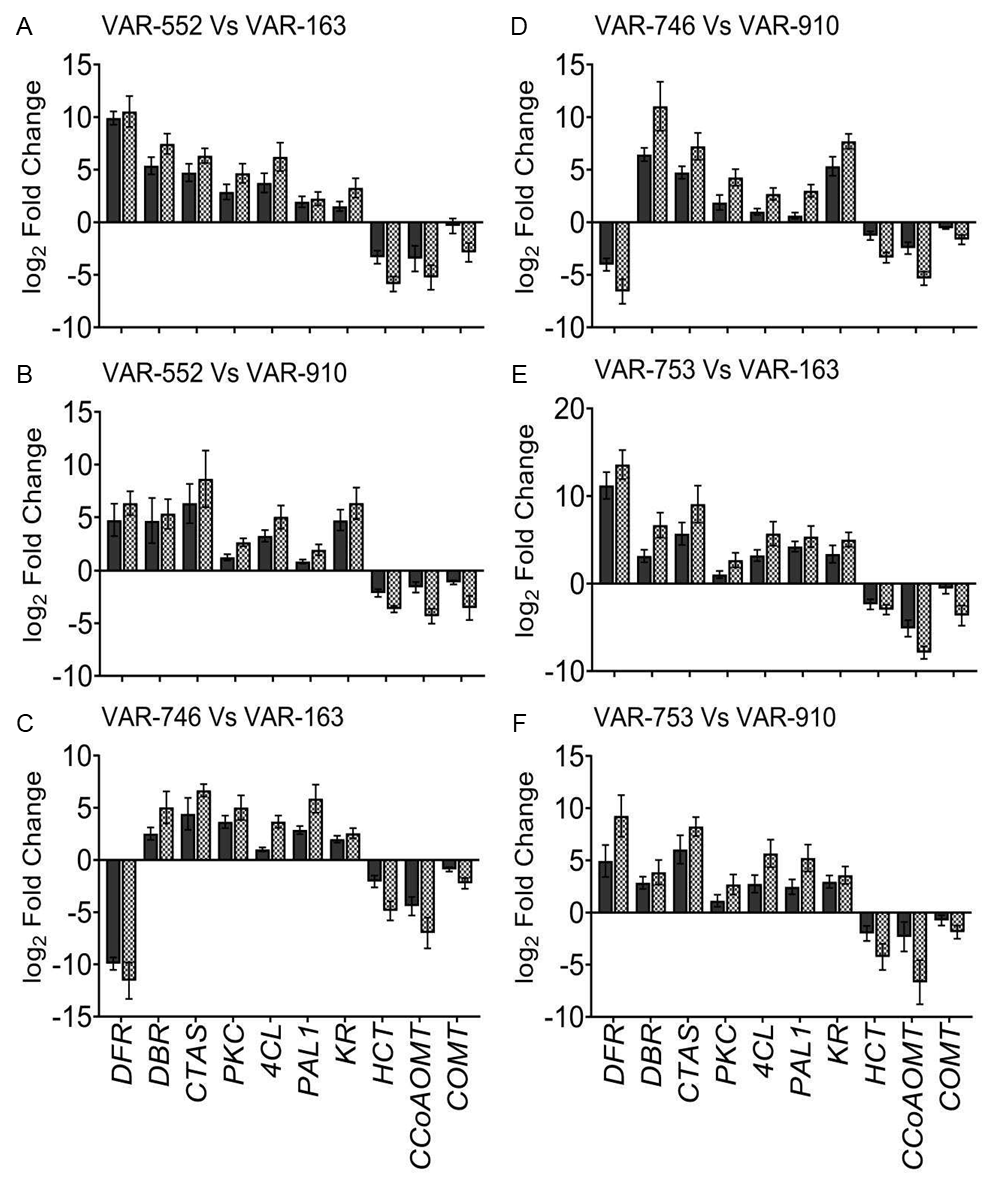


**Fig S4: Validation of the expression of candidate genes by means of quantitative real-time PCR (qRT-PCR).** (A-F) The expression of genes involved in the metabolic pathways associated with PD biosynthesis while comparing accessions (A) VAR-552 and VAR-163, (B) VAR-552 and VAR-910, (C) VAR-746 and VAR-163 and (D) VAR-746 and VAR-910, (E) VAR-753 and VAR-163 and (F) VAR-753 and VAR-910. Ten representative genes namely DFR, DBR, CTAS, PKC, 4CL, PAL1, KR, HCT, CCoAOMT and COMT were selected to validate the expression of genes obtained from RNAseq using RT-qPCR. Bars indicate mean ± SE (n=3) of log2 fold-change obtained from the respective experiments. Positive values represent upregulation and negative values represent downregulation. The gene GADPH was used as an internal reference gene. Black bars show log2 fold change of RNASeq data and hatched bars show the log2 fold change of qRT-PCR.


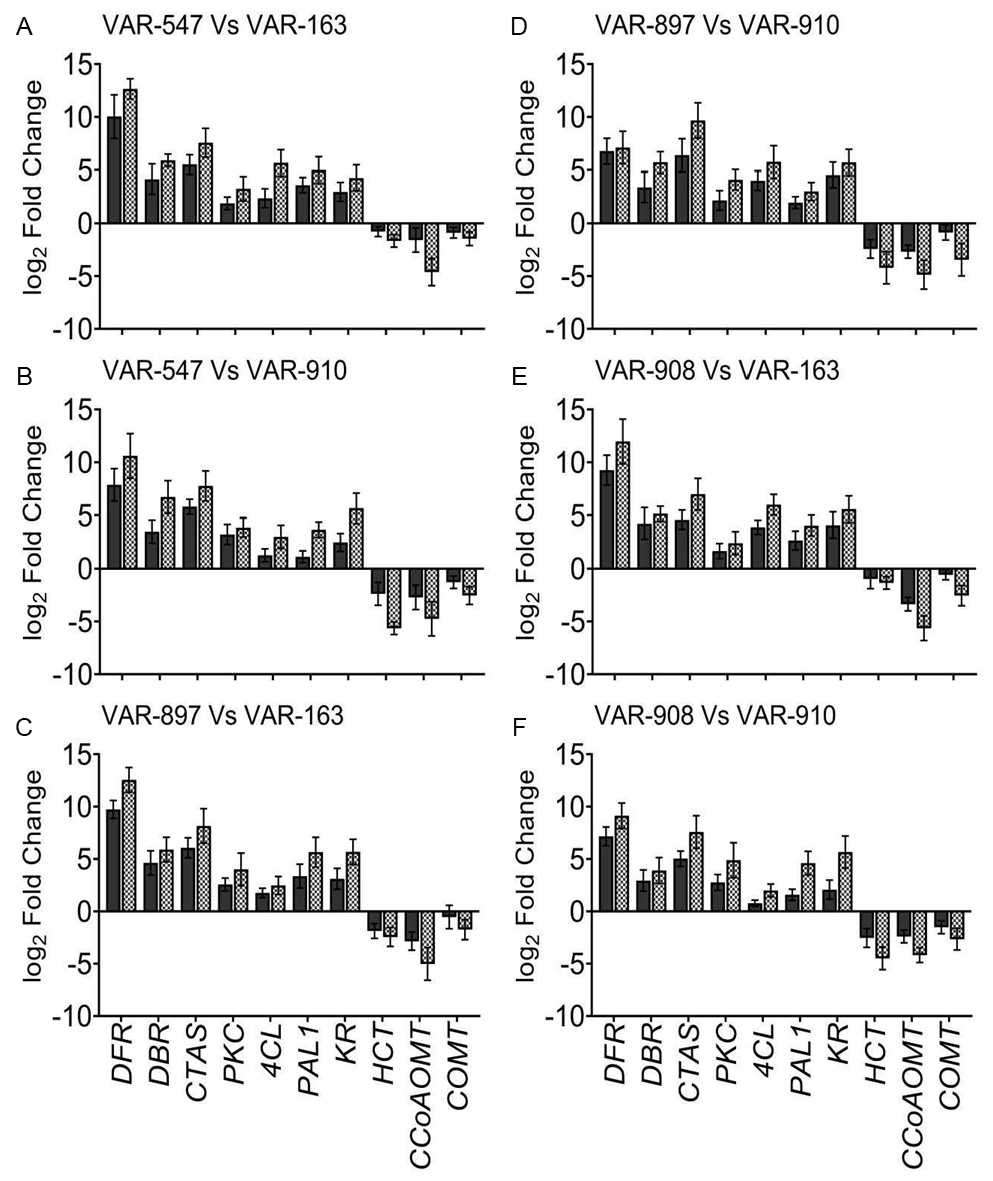


**Fig S5: Validation of the expression of candidate genes by means of quantitative real-time PCR (qRT-PCR).** (A-F) The expression of genes involved in the metabolic pathways associated with PD biosynthesis while comparing accessions (A) VAR-547 and VAR-163,(B) VAR-547 and VAR-910, (C) VAR-897 and VAR-163 and (D) VAR-897 and VAR-910, (E)VAR-908 and VAR-163 and (F) VAR-908 and VAR-910. Ten representative genes namely DFR, DBR, CTAS, PKC, 4CL, PAL1, KR, HCT, CCoAOMT and COMT were selected tovalidate the expression of genes obtained from RNAseq using RT-qPCR. Bars indicate mean ± SE (n=3) of log2 fold-change obtained from the respective experiments. Positive values represent upregulation, and negative values represent downregulation. The gene GADPH was used as an internal reference gene. Black bars show log2 fold change of RNASeq data and hatched bars show the log2 fold change of qRT-PCR.

**Table S1:** **List of H. macrophylla accessions used for primary screening.** Green filled cells represent the 13 accessions selected for experiments*.*

| VAR-0908_000 (VAR 908) | VAR-0553_001 (VAR 553) | VAR-0897_000 (VAR 897) |
| --- | --- | --- |
| VAR-0553_002 | VAR-0552_000 (VAR 552) | VAR-0552_001 |
| VAR-0828_000 | VAR-0766_001 | VAR-0158_000 |
| VAR-0547_000 (VAR-547) | VAR-0542_000 | VAR-0536_000 |
| VAR-0749_000 | VAR-0556_000 | VAR-0912_000 |
| VAR-0765_000 | VAR-0906_000 | VAR-0583_000 |
| VAR-0556_001 | VAR-0836_000 | VAR-0571_000 |
| VAR-0844_000 | VAR-0535_000 | VAR-0576_000 |
| VAR-0879_001 | VAR-0768_000 | VAR-0827_001 |
| VAR-0827_000 (VAR 827) | VAR-0556_002 | VAR-0539_000 |
| VAR-0582_000 | VAR-0562_000 | VAR-0561_000 |
| VAR-0751_000 (VAR 751) | VAR-0580_000 | VAR-0560_000 |
| VAR-0574_000 | VAR-0260_002 | VAR-0763_000 |
| VAR-0550_000 | VAR-0575_000 | VAR-0561_001 |
| VAR-0750_000 | VAR-0747_000 | VAR-0573_000 |
| VAR-0566_000 | VAR-0921_000 | VAR-0446_000 |
| VAR-0846_000 | VAR-0547_002 | VAR-0567_000 |
| VAR-0555_000 | VAR-0564_000 | VAR-0706_000 |
| VAR-0915_000 | VAR-0132_002 | VAR-0438_000 |
| VAR-0746_000 (VAR 746) | VAR-0768_001 | VAR-0569_000 |
| VAR-0750_001 | VAR-0212_001 | VAR-0843_000 |
| VAR-0009_000 | VAR-0922_001 | VAR-0856_000 |
| VAR-0097_000 | VAR-0837_000 | VAR-0452_000 |
| VAR-0146_000 | VAR-0440_000 | VAR-0163_000 (VAR 163) |
| VAR-0579_000 | VAR-0543_000 | VAR-0823_000 |
| VAR-0771_000 | VAR-0260_000 | VAR-0538_000 |
| VAR-0758_000 | VAR-0554_000 | VAR-0913_000 |
| VAR-0551_000 | VAR-0754_000 | VAR-0290_000 |
| VAR-0755_000 | VAR-0745_000 | VAR-0565_000 |
| VAR-0908_000 (VAR 908) | VAR-0553_001 (VAR 553) | VAR-0897_000 (VAR 897) |
| VAR-0553_002 | VAR-0552_000 (VAR 552) | VAR-0552_001 |
| VAR-0828_000 | VAR-0766_001 | VAR-0158_000 |
| VAR-0547_000 (VAR-547) | VAR-0542_000 | VAR-0536_000 |
| VAR-0749_000 | VAR-0556_000 | VAR-0912_000 |
| VAR-0765_000 | VAR-0906_000 | VAR-0583_000 |
| VAR-0556_001 | VAR-0836_000 | VAR-0571_000 |
| VAR-0844_000 | VAR-0535_000 | VAR-0576_000 |
| VAR-0879_001 | VAR-0768_000 | VAR-0827_001 |
| VAR-0827_000 (VAR 827) | VAR-0556_002 | VAR-0539_000 |
| VAR-0582_000 | VAR-0562_000 | VAR-0561_000 |
| VAR-0751_000 (VAR 751) | VAR-0580_000 | VAR-0560_000 |
| VAR-0574_000 | VAR-0260_002 | VAR-0763_000 |
| VAR-0550_000 | VAR-0575_000 | VAR-0561_001 |
| VAR-0750_000 | VAR-0747_000 | VAR-0573_000 |
| VAR-0566_000 | VAR-0921_000 | VAR-0446_000 |
| VAR-0846_000 | VAR-0547_002 | VAR-0567_000 |
| VAR-0555_000 | VAR-0564_000 | VAR-0706_000 |
| VAR-0915_000 | VAR-0132_002 | VAR-0438_000 |
| VAR-0746_000 (VAR 746) | VAR-0768_001 | VAR-0569_000 |
| VAR-0750_001 | VAR-0212_001 | VAR-0843_000 |
| VAR-0009_000 | VAR-0922_001 | VAR-0856_000 |
| VAR-0097_000 | VAR-0837_000 | VAR-0452_000 |
| VAR-0146_000 | VAR-0440_000 | VAR-0163_000 (VAR 163) |
| VAR-0579_000 | VAR-0543_000 | VAR-0823_000 |
| VAR-0771_000 | VAR-0260_000 | VAR-0538_000 |
| VAR-0758_000 | VAR-0554_000 | VAR-0913_000 |
| VAR-0551_000 | VAR-0754_000 | VAR-0290_000 |
| VAR-0755_000 | VAR-0745_000 | VAR-0565_000 |

**Table S2: Transitions of ions used for quantification of metabolites.** The transition of ions used for quantification and mode of ionizations, QIT, collision energies (CE) of corresponding transition in eV and retention time (t_R_) in minutes of the 14 metabolites analyzed in leaf extracts of Hydrangea accessions.

| **Sl.no** | **Molecule** | **QIT** | **CE (eV)** | **t_R_ (min)** | **Ionization** |
| --- | --- | --- | --- | --- | --- |
| 1 | Phyllodulcin | 287.1→269.1 | 13 | 4.036 | Positive |
| 2 | Hydrangenol | 257.1→239.0 | 13 | 3.933 | Positive |
| 3 | Naringenin | 273.1→152.9 | 25 | 2.460 | Positive |
| 4 | Resveratrol | 229.1→135.0 | 17 | 2.777 | Positive |
| 5 | Umbelliferone | 163.0→118.9 | 17 | 2.093 | Positive |
| 6 | Caffeic acid | 179.0→135.0 | 14 | 1.296 | Negative |
| 7 | Ferulic acid | 193.1→134.1 | 18 | 2.194 | Negative |
| 8 | trans-Cinnamic acid | 147.0→103.1 | 10 | 3.134 | Negative |
| 9 | *p*-Coumaric acid | 163.0→119.0 | 14 | 1.952 | Negative |
| 10 | Scopolin | 355.1→193.0 | 9 | 0.926 | Positive |
| 11 | Scopoletin | 193.1→133.0 | 21 | 2.160 | Positive |
| 12 | Esculin | 341.1→179.0 | 13 | 0.611 | Positive |
| 13 | Esculetin | 179.1→122.9 | 25 | 1.238 | Positive |
| 14 | Fraxetin | 209.5→149.0 | 21 | 1.738 | Positive |

**Table S3:** List of qRT-PCR primers used for validation of identified differentially expressed genes via RNA sequencing

| **Gene** | **Forward primer**  **5’→3’** | **Reverse primer**  **5’→3’** |
| --- | --- | --- |
| Glyceraldehyde-3-  phosphate dehydrogenase -  *GADPH* | GGCTGAGACTGGAGCGGAAT | CAAACATGGGGGCGTCTTTGC |
| Double Bond Reductase -  *DBR* | ACCGTTGTGCCTTACATCAG | ACGGCCACTAAAGAGTCCAA |
| Dihydroflavonol 4-reductase  - *DFR* | ATGTTGGACGCCTCTCCATC | CACAAGTCCACACCCAAGAG |
| p-Coumaroyl tri acetic acid  synthase - *CTAS* | CAGGCAAAAGTGGGTCTGAA | GAAAAACACACATGCGCTGG |
| Polyketide Cyclase - PKC | TGCAGCCCATTCCAAATCAG | TGATGAAGGACCTTTCGGGA |
| Phenylalanine ammonia-  lyase 1 – *PAL1* | ATCAGAGAGTGCCGGTCTTT | ACCGGACTTTCTCTCCTGTC |
| 4-coumarate CoA ligase –  *4CL* | TGGTTCCAAGATCTCCGAGG | GAGCTTTTGGGATTGCGTCT |
| Keto reductase - *KR* | GGTAGCCCTGAATATGTGCG | GTCGACACGATGTGGGTAGT |
| Hydroxycinnamoyl  transferase - *HCT* | AGATGGACTTTCAGCCCTCC | GAGGATGGTCCGATCGATGA |
| Caffeoyl-CoA 3-O-  methyltransferase -  *CCoAOMT* | GGCATGGAGCACAAGATCAA | GGGCTTGTCAGCGTCAATAA |
| Caffeic acid O-  methyltransferase - *COMT* | GTGTCGATCGGAAGGCCATA | ATGCGACAGCACAAGGTAAC |

**Supplementary Protocols**

**Supplementary protocol S1. RNA isolation, sequencing and analysis:**

To isolate total RNA from the plant tissues, the Spectrum™ Plant Total RNA Kit, obtained from Sigma Aldrich, was employed following the manufacturer's guidelines, and it was found to be highly effective for isolating RNA from Hydrangea tissues. Total RNA was isolated and analyzed from three replicates of 14 selected accessions, each with varying levels of PD and HD concentration. The quality of RNA was assessed using NanoDrop™ 2000c spectrophotometry from Thermo Scientific™, with a 10 µL volume collected and used for sequencing. RNA integrity was further evaluated using the RNA Nano 6000 Assay Kit on the Bioanalyzer 2100 system by Agilent Technologies in California, USA. For cDNA library preparation and transcriptome sequencing, Novogene conducted the procedures using the NEBNext® UltraTM RNA Library Prep Kit for Illumina® (NEB, USA). Initially, mRNA was purified from total RNA using poly-T oligo-attached magnetic beads, followed by fragmentation. Subsequently, the first and second strands of cDNA were synthesized using a random hexamer primer and M- MuLV Reverse Transcriptase (RNase H-) along with DNA Polymerase I and RNase H, respectively. cDNA fragments of lengths between 370 to 420 bp were selected after purifying library fragments using the AMPure XP system from Beckman Coulter in Beverly, USA. PCR was then carried out with Phusion High-Fidelity DNA polymerase, Universal PCR primers, and Index (X) Primer. The quality of the libraries was assessed using the Agilent Bioanalyzer 2100 system. Upon library preparation, 150 bp pair end reads were generated after clustering. The reference genome and gene model annotation files were downloaded directly from https://plantgarden.jp. Indexing of the reference genome and read alignment to the reference genome were performed using Hisat2 v2.0.5. The mapped reads from each sample were assembled using StringTie (v1.3.3b) (Pertea et al, 2015) in a reference-based approach. FeatureCounts v1.5.0-p3 was utilized to count the number of reads mapped to each gene. Differential expression analysis was conducted using the DESeq2 R package (v1.20.0). The resulting p-values were adjusted using the Benjamini and Hochberg’s approach to control the False Discovery Rate (FDR), represented as padj value. Significantly differentially expressed genes were identified with a corrected p-value of 0.05 and an absolute fold change of 2 as the threshold. To gain insights into the functional significance of differentially expressed genes, Gene Ontology (GO) enrichment analysis was performed using the clusterProfiler R package, with GO terms exhibiting corrected p-values below 0.05 considered significantly enriched by the differentially expressed genes. Additionally, KEGG (Kyoto Encyclopedia of Genes and Genomes) pathway enrichment analysis was conducted to assign each gene to its respective pathways^25,26^.

**Supplementary protocol S2. Detailed description of qRT-PCR method:**

The RevertAid First Strand cDNA Synthesis Kit from Thermo Scientific was utilized to perform cDNA synthesis, and 0.3 µg of total RNA was employed as the starting material. The synthesis was initiated using oligo(dT) primers. The primers for the quantitative real-time polymerase chain reaction (qRT-PCR) were created through primer design software Primer3, and they were subsequently synthesized by the company Metabion (Germany). The list of primers designed for validation with qRT- PCR are represented in Table S1. Specific criteria during the primer design process included achieving a melting temperature (Tm) within the range of 60±1°C, designing primers with a length between 18 and 25 base pairs, preferably located close to the 3'- end of the target sequence, and ensuring a GC content between 40% and 60%. This strategy aimed to generate PCR products that are unique and relatively short, ranging from 60 to 150 base pairs in length. Gene expression level was employed using qPCR (TouchTM system, Bio-Rad. For the qPCR reactions, the iQ SYBR Green Supermix from Bio-Rad (Hercules, CA, USA) was used. The samples underwent examination in triplicates, following this procedure: an initial activation cycle was carried out for 3 minutes at 95°C, accompanied by 40 amplification cycles, with each cycle comprising 15 seconds at 95°C and then 30 seconds at either 58°C or 60°C. Subsequently, a single melting curve cycle was executed, spanning from 65°C to 95°C, with increments of 0.5°C every 5 seconds. The Ct (Cycle threshold) values obtained were exported from the CFX Software (Bio-Rad Laboratories) and were employed to calculate the efficiency of PCR amplification. Normalization factors were calculated using glyceraldehyde-3- phosphate dehydrogenase (GAPDH) as reference gene, which has also been used in previous studies ^40^. PCR amplification efficiency was determined following the guidelines outlined in prior instructions ^41^. Only experiments exhibiting an efficiency falling within the range of 90% to 110% were considered. Normalization factors were calculated using geNORM ^42^. The expression levels of genes were represented as fold change.
